# Inhibition of keratinocyte necroptosis mediated by RIPK1/RIPK3/MLKL provides a protective effect against psoriatic inflammation

**DOI:** 10.1038/s41419-020-2328-0

**Published:** 2020-02-19

**Authors:** Xiaoru Duan, Xinxin Liu, Nian Liu, Yuqiong Huang, Zilin Jin, Song Zhang, Zhangyin Ming, Hongxiang Chen

**Affiliations:** 1 Department of Dermatology, Union Hospital, Tongji Medical College, Huazhong University of Science and Technology, 430022 Wuhan, China; 2 Department of Pharmacology, Tongji Medical College, Huazhong University of Science and Technology, 430030 Wuhan, China; 3 Department of Dermatology, Union Shenzhen Hospital, Huazhong University of Science and Technology, 518052 Shenzhen, China

**Keywords:** Necroptosis, Psoriasis, Chronic inflammation

## Abstract

Psoriasis is a common autoimmune and chronic inflammatory skin disorder globally affecting 0.51–11.43% of adults. Inflammation-associated cell death in keratinocytes plays a key role in the process of integrate inflammatory cascade in psoriasis. Necroptosis is a regulated necrotic cell death mediated by receptor interacting protein kinase 1 (RIPK1), RIPK3, and mixed lineage kinase domain-like pseudokinase (MLKL), which participates in many human inflammatory diseases. However, the mechanism and function of programmed necrosis in psoriasis is not well-illustrated. In the current study, we provide evidence for the involvement of necroptosis in psoriasis. RIPK1 and MLKL were significantly upregulated and localized in all layers of the epidermis in human psoriatic lesions, while RIPK3 and phosphorylated MLKL were mainly expressed in keratinocytes, which located in the upper layers. Increased tendency of necroptosis was also found in IMQ-induced psoriasiform skin of mice. Further, we discovered that both the inhibitor of RIPK1 R-7-Cl-O-Necrostatin-1 (Nec-1s) and MLKL-inhibitor necrosulfonamide (NSA) suppressed necroptosis in HaCaT cells and IMQ mouse models, powerfully blocked IMQ-induced inflammatory responses in vivo, and significantly downregulated the production of inflammatory factors like IL-1β, IL-6, IL-17A, IL-23a, CXCL1, and CCL20. These findings promote the development of new therapies for the treatment of necroptosis-activated pathologies for psoriasis.

## Introduction

Psoriasis is a common autoimmune and chronic inflammatory skin disorder portrayed as hyperproliferation and maturation impairment of keratinocytes, increased immune cells infiltration and blood vessel formation, and accumulation in proinflammatory cytokines^1^. Globally, the prevalence of psoriasis in adults is 0.51–11.43%^2^. Psoriasis intrinsic defects exacerbate the imbalance between anti-inflammatory and proinflammatory signals in an autocrine or paracrine manner that contributes to various aspects of its pathogenesis^3^. Keratinocytes play a pivotal role in the early stage of psoriasis and in the maintenance of chronic course. A recent study provided compelling evidence that keratinocytes are critical for the development of complete psoriasis inflammatory cascade^4^. Destruction of the epidermal barrier of psoriasis makes keratinocytes more susceptible to various external harmful substances, causing cell damage or even cell death.

Necroptosis, also known as programmed necrosis, is a regulated necrotic cell death pattern which was first proposed in 2005^5^ involving receptor interacting protein kinase 1 (RIPK1), RIPK3 and mixed lineage kinase domain-like pseudokinase (MLKL). A number of different stimulations can initiate necroptosis^6^, including cytokines, viral infection, chemicals, or damage-associated molecular patterns (DAMPs), and so on. Tumor necrosis factor-alpha (TNF-α) is a cytokine, which is currently the most intensively and extensively studied stimuli and probably the most important trigger for necroptosis^7^, and it is also considered the main cytokine in patients with psoriasis. In cells stimulated by TNFα, activation of RIPK1 kinase activity plays a crucial role in determining whether a cell is headed for necroptosis or apoptosis^8^. When blocking the formation of complex IIa consist of RIPK1, FADD, and caspase-8, activated RIPK1 interacts with RIPK3 through the RIP homotypic interaction motif (RHIM) to form complex IIb and mediate phosphorylation of MLKL. Phosphorylated MLKL (pMLKL) acts as an executor to ultimately induce necroptosis, which can be penetrated into the plasma membrane and organelles causing membrane rupture and release of their contents as DAMPs^7^.

Necroptosis can strongly facilitate inflammation by mediating the manufacture of inflammatory cytokines and disrupting the release of DAMPs, involved in the development of various diseases^9^ such as multiple sclerosis, myocardial ischemia-reperfusion injury, inflammatory bowel disease, and some dermatitis. Bonnet et al.^10^ found that programmed necrosis promotes skin inflammation in apoptosis-associated protein FADD-specific knockout mice. Caspase-8 specific knockout mice can also cause inflammatory lesions in the skin, and further knockout RIP3 prevent inflammation in these mice^11^. Mouse models confirm that keratinocytes necroptosis can effectively induce severe cutaneous adverse drug reactions^12^. The above studies suggest that necroptosis in keratinocytes may participate in the pathogenesis of human inflammatory cutaneous diseases.

Unfortunately, the mechanism and function of programmed necrosis in psoriasis is poorly understood and contradictory opinions existed in the studies^13–15^. In this study, the expression levels of necroptosis-related molecules RIPK1, RIPK3, and MLKL were reported to be increased in psoriatic lesions, especially in keratinocytes of the epidermis while similar tendency was found in imiquimod (IMQ) -induced psoriasiform skin of mice, which is the most widely used mouse model for studying human psoriasis^16^. To further explore whether programmed necrosis in keratinocytes is involved in psoriatic skin inflammation, R-7-Cl-O-Necrostatin-1 (Nec-1s)^17^ was introduced as an improved inhibitor of RIPK1 and necrosulfonamide (NSA)^18^ as the specific inhibitor of MLKL. The two inhibitors are safe and effective on blocking necroptosis in many several mouse models of human diseases, such as ischemic brain, multiple sclerosis, and Alzheimer’s disease^9^. In this study, they displayed their ability to suppress necroptosis in HaCaT cells and IMQ mouse models, powerfully block IMQ-induced inflammatory responses in vivo, and significantly downregulated the production of inflammatory factors like IL-1β, IL-6, IL-17A, IL-23a, CXCL1, and CCL20. These findings highlight the proinflammatory effect of necroptosis in the pathogenesis of psoriasis, and the inhibition of necroptosis as a promising method for the treatment of psoriasis.

## Results

### Increased expression of necroptosis-related hallmarks in the epidermis of human psoriasis lesions

**Fig. 1:**
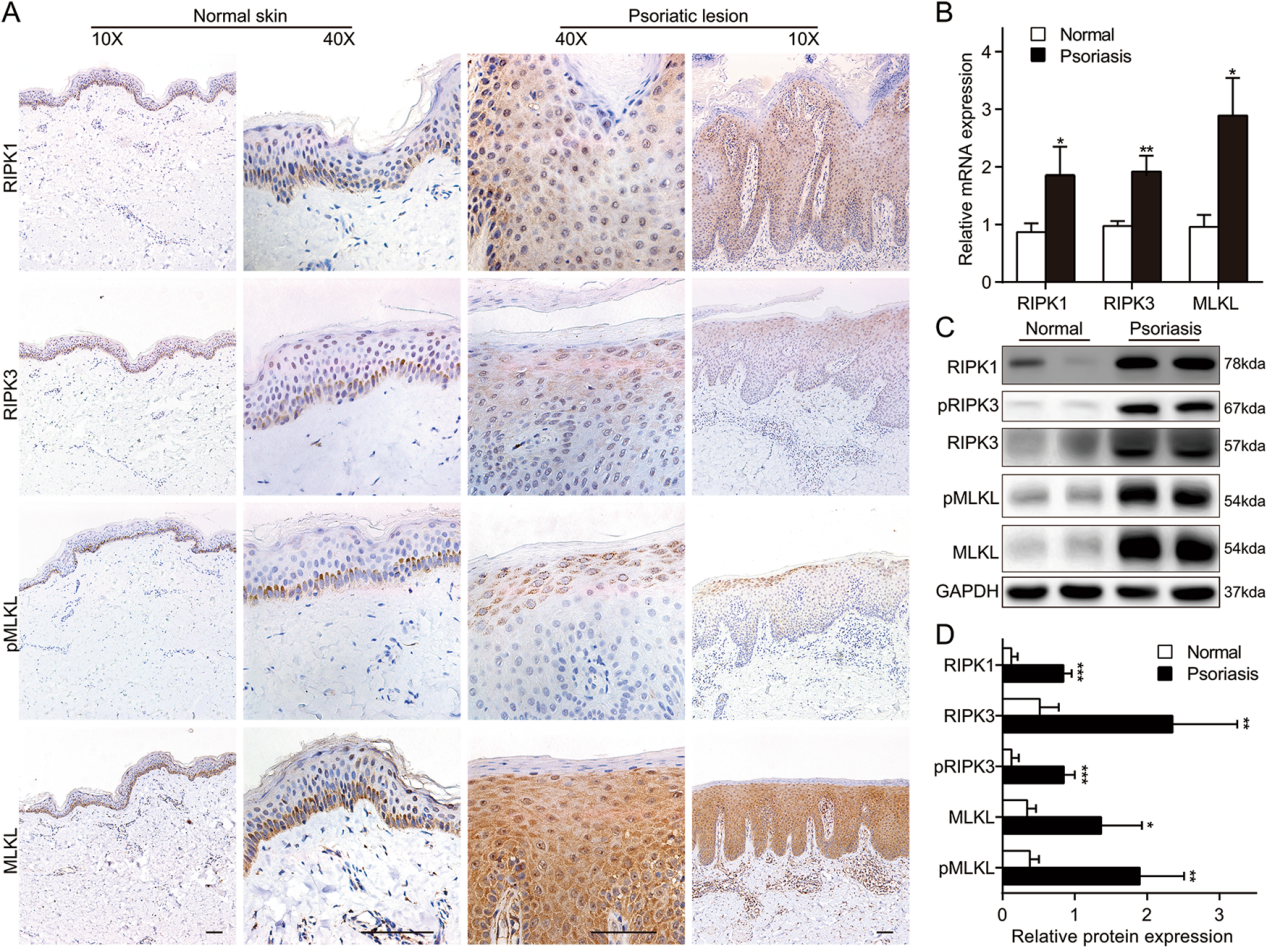
Expression of necroptosis-related genes in the epidermis of human psoriasis lesions. **a** Samples from normal skin and psoriatic lesions were stained with RIPK1, RIPK3, MLKL, and pMLKL(S358). Representative images are shown. Scale bar represents 200 μm. **b** mRNA levels of RIPK1, RIPK3, and MLKL in skin tissues from the normal and psoriasis groups were analyzed by real-time quantitative PCR. **c**, **d** Protein levels of RIPK1, RIPK3, pRIPK3(S227), MLKL and pMLKL (S358) were quantified by western blotting. GAPDH was used as a loading control. Relative protein expression was normalized to that of the internal control. Results are representative of three independent experiments. Error bars (**b**, **d**) represent mean ± standard deviation (SD). *p < 0.05, **p < 0.01, and ***p < 0.001 when compared.

Although increased expression of pRIPK1 and RIPK3 in the epidermis of human psoriasis samples compared to nonlesional skin has been observed by immunohistochemistry^15,19^, there is a lack of convincing evidence supporting the activity of necroptosis in human psoriasis. More importantly, other necroptosis-related features, such as the role of MLKL and pMLKL in psoriatic lesions, are unclear. The expression of necroptosis-related genes was analyzed in skin lesions from seven psoriasis patients and several samples from healthy adult skin by immunohistochemistry. In healthy adult epidermis, RIPK1, RIPK3, MLKL, and pMLKL were expressed in the basal layer of the epidermis in normal skin, whereas in human psoriatic skin lesions, RIPK1 and MLKL were significantly expressed in in all layers of the epidermis peculiarly with the strongest expression of MLKL, while RIPK3 and pMLKL were mainly expressed in the upper layers (Fig. 1a). To further confirm the immunohistology outcomes, the transcription and protein expression levels of necroptosis-related genes in psoriatic lesions were explored. The level of MLKL in psoriatic lesions was approximately three times that of the normal group. Similarly, there was significant up-regulation of RIPK1 and RIPK3 in psoriasis tissues compared to normal samples (Fig. 1b). Figure 1c shows the protein levels of RIPK1, RIPK3, phosphorylated RIPK3 (pRIPK3), MLKL, pMLKL, and GAPDH in two sets of samples. Semi-quantitative western blot analysis showed that RIPK1, RIPK3, pRIPK3, MLKL, and pMLKL levels were significantly higher in psoriatic group than in the normal control group (Fig. 1d). Consistent with the findings in human psoriatic lesions, real-time quantitative PCR (qRT-PCR) and western blotting revealed an increase in expression levels of RIPK1, RIPK3, MLKL, and pMLKL in IMQ-induced psoriasiform skin of mice (Fig. S1). These data showed dysregulation of necroptosis-related markers in the epidermis of the psoriasis.

### Inhibition of RIPK1 attenuated RIPK1/RIPK3-mediated necroptosis in vitro

Key necroptosis-related molecules were highly expressed in the epidermis of psoriasis lesions, suggesting that keratinocytes experienced necroptosis. To mimic necroptosis keratinocytes in psoriatic lesions, we applied the most commonly used necroptosis inducing treament^20^ in the immortalized human keratinocytes HaCaT cells^21^, which includes a mixture of TNF-α (T), Smac (S), and the pan-caspase inhibitor z-VAD-fmk (Z), hereinafter referred to as TSZ. The cell viability of TSZ-induced programmed necrosis in HaCaT cells at different hours by CCK-8 and lactate dehydrogenase (LDH) release rate was examined (Fig. S2A–B). The point at which cell mortality was highest was considered to be at 8 h. Besides, pMLKL is an execution marker of necroptosis and HMGB1 is one of the most common DAMPs, which can be released either passively by necrotic cells^22^. As shown in Fig. S2C, enhanced levels of pMLKL and HMGB1 were observed at 2 h, and prominent expression occurred at 8 h, which was selected as the induction time in subsequent experiments.

**Fig. 2:**
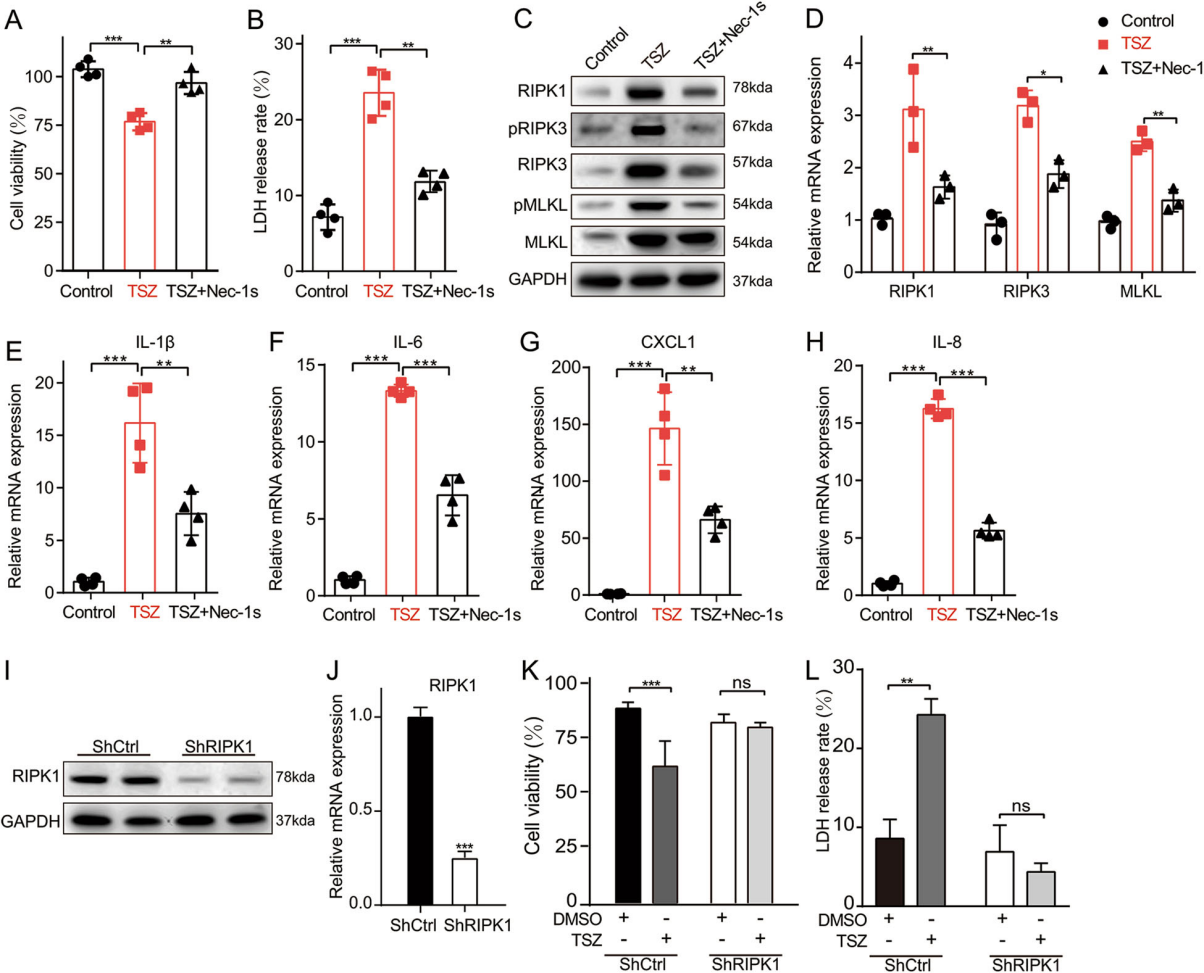
Inhibition of RIPK1 attenuated RIPK1/RIPK3- mediated necroptosis in vitro. **a**–**h** HaCaT cells were treated with TNF-α (100 ng/mL), Smac (100 nM), and z-VAD-fmk (20 μM) for 8 h (hereafter abbreviated as TSZ) to induce necroptosis and then stimulated with Nec-1s (10 μM). **a** Cell viability was measured by the Cell Counting Kit-8 (CCK-8) assay. **b** LDH release was measured by the cytotoxicity LDH assay kit. **c** Protein levels of RIPK1, RIPK3, pRIPK3(S227), MLKL and pMLKL (S358) were quantified by western blotting. GAPDH served as the loading control. **d** mRNA level of RIPK1, RIPK3, and MLKL was determined by real-time quantitative PCR in the three groups. **e**–**h** Real-time quantitative PCR was performed to analyze the mRNA level of IL-1β (**e**), IL-6 (**f**), CXCL1 (**g**), and IL-8 (**h**). **i**, **j** Knockdown of RIPK1 by shRNA in HaCaT cells was confirmed by western blotting (**i**) and real-time quantitative PCR (**j**). **k**, **l** After RIPK1-knockdown in HaCAT cells, they were treated with TSZ. Cell viability was measured by the Cell Counting Kit-8 (CCK-8) assay. LDH release was measured by the cytotoxicity LDH assay kit. Error bars represent mean ± standard deviation (SD). ns *p* > 0.05, *p < 0.05, **p < 0.01, and ***p < 0.001 when compared. All the assays were repeated three times and the results were consistent.

Treatment with the highly RIPK1 specific kinase inhibitor Nec-1s^17^ prevented TSZ-induced necroptosis in HaCaT cells, accompanied by the resumption of cell viability and suppression of LDH release (Fig. 2a, b). To investigate the molecular mechanism of Nec-1s mediated inhibition of necroptosis, the major steps of necroptotic signal transduction including phosphorylation of RIPK3 and MLKL were examined. As shown in Fig. 2c, d, the expression levels of RIPK1, RIPK3, and MLKL were downregulated by Nec1-1s, while the pRIPK3 and pMLKL were also reduced. To further explore the anti-inflammation effects of Nec-1s in HaCaT cells, qRT-PCR was executed to analyze the mRNA expression of inflammatory cytokines including IL-1β, IL-6, CXCL1, and IL-8, which was significantly decreased after Nec-1s stimulation (Fig. 2e–h). Since RIPK1 is a critical mediator for TSZ-induced necrosis, knockdown of RIPK1 also almost terminated this form of cell death (Fig. 2i–l). Collectively, inhibition of RIPK1 blocked the RIPK1/RIPK3-dependent necroptosis and significantly reduced the expression of inflammatory cytokines in vitro.

### Treatment with necroptosis inhibitor Nec-1s reduced inflammation in mice with IMQ-induced psoriasiform dermatitis

**Fig. 3:**
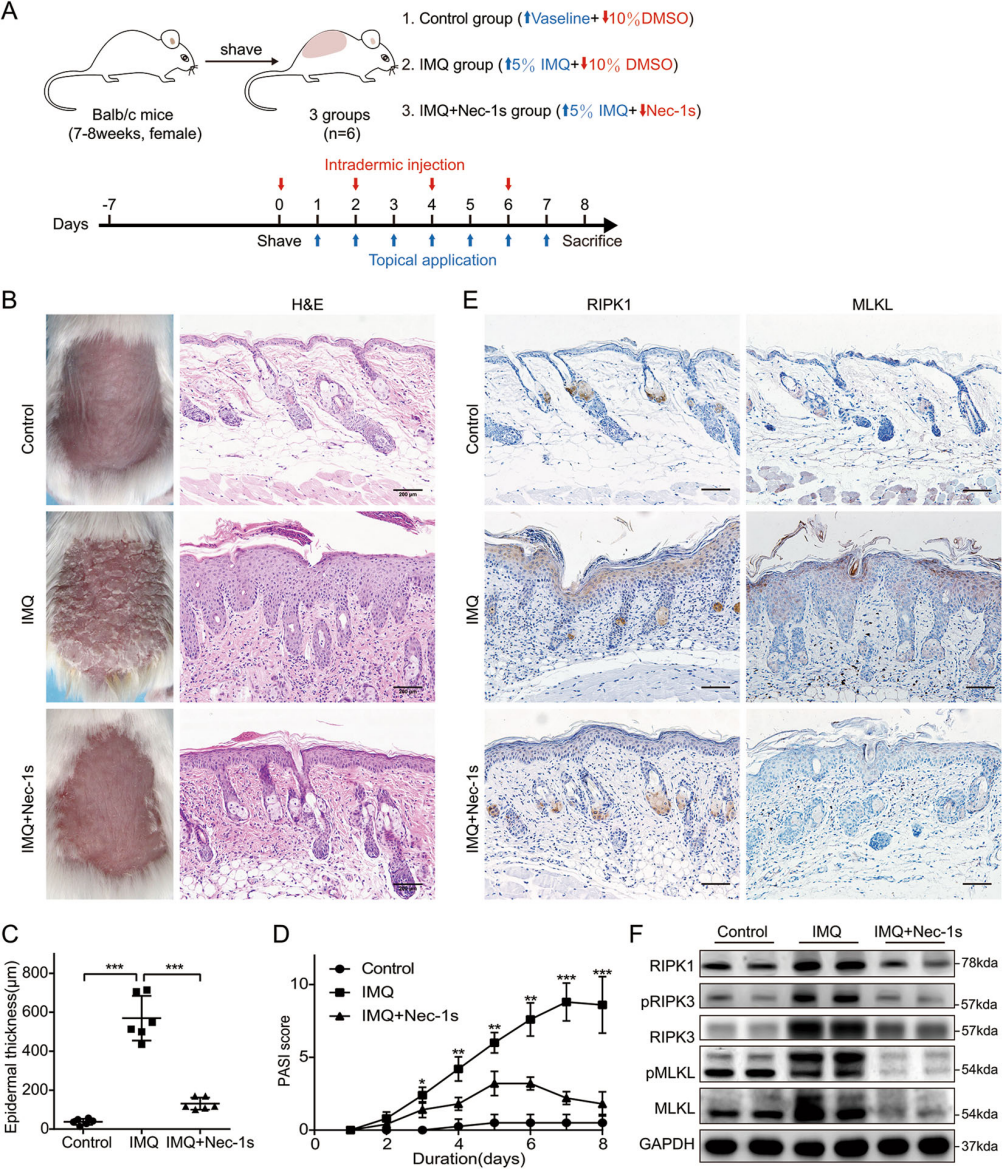
Nec-1s improved the morphological and histological features and the necroptosis-related protein expression in mice with IMQ-induced psoriasiform dermatitis. **a** Schematic diagram of the animal experiment protocol for the Control, IMQ, and IMQ + Nec-1s groups (*n* = 6). **b** Representative macroscopic views and H&E staining of cross-sectional slices of the dorsal skin of BALB/c mice on the 8th day. Scale bar represents 200 μm. **c** The epidermal thickness of the dorsal skin on the 8th day was measured by four randomly selected fields per section of each mouse. **d** Daily assessment of epidermal erythema, scales, and thickening of the dorsal skin. The PASI score was calculated by adding the scores of three independent criteria (ranging from 0 to 12). **e** Immunohistochemical staining for RIPK1 and MLKL in the dorsal mouse skin samples for the three groups. Representative images are shown. Scale bar represents 200 μm. **f** Protein levels of RIPK1, pRIPK3(S232), RIPK3, MLKL and pMLKL (S345) were quantified by western blotting. GAPDH served as the loading control. Error bars represent mean ± standard deviation (SD). *p < 0.05, **p < 0.01, and ***p < 0.001 when compared. All assays were repeated three times with consistent results.

To evaluate whether necroptosis signals contribute to psoriasis inflammation, we induced a mouse model of psoriasis dermatitis with topical IMQ application^4^ and simultaneously conducted intradermal injection of Nec-1s or DMSO as control every other day (Fig. 3a). By day 3, the IMQ treated mice showed skin inflammation signs of erythema, scales and increased skin thickness compared to the control group, and the lesions gradually worsened with extended IMQ application. Macroscopic findings revealed that pretreatment with Nec-1s significantly attenuated clinical phenotype of IMQ-induced psoriasiform dermatitis (Fig. 3b). Hematoxylin-eosin (HE) staining performed on the dorsal skin of the mice on day 8, revealed significant epidermal hyperplasia, significantly increased acanthosis, parakeratosis, telangiectasia, and inflammatory cell infiltration in the IMQ group. Nec-1s significantly improved the histologic changes caused by IMQ, showing less parakeratosis and less epidermal thickening than in the IMQ group (Fig. 3b). The skin thickness of the IMQ group was significantly increased compared with the normal group, while Nec-1s group was decreased (Fig. 3c). The average Psoriasis Area and Severity Index (PASI) score of three groups were graded daily to assess their clinical changes, as plotted in Fig. 3d. There was no significant change in cumulative PASI scores of the control group. Interestingly, PASI scores were lower in the IMQ + Nec-1s group than in the IMQ group. From day 4, the differences in the scores were statistically significant.

**Fig. 4:**
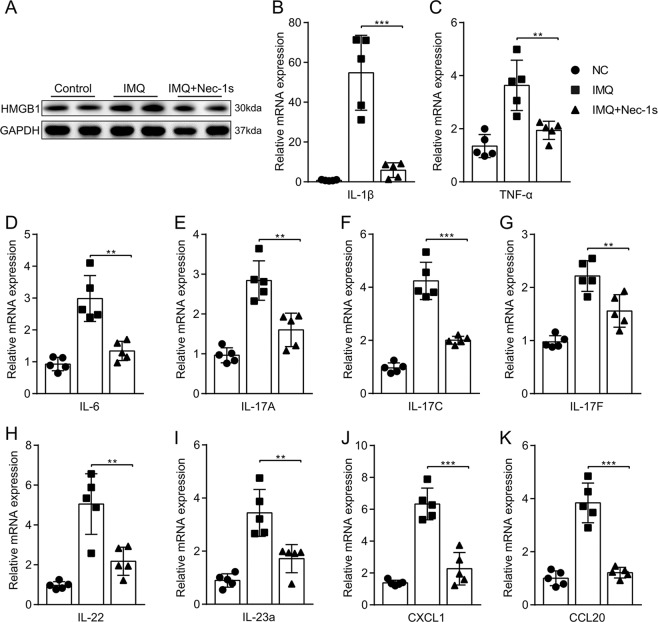
Nec-1s reduced the expression levels of DAMPs and proinflammatory cytokines except IFN in mice with IMQ-induced psoriasiform dermatitis. **a** Protein level of HMGB1 was quantified by western blotting in the three groups of mice. **b**–**m** Real-time quantitative PCR was performed to quantify the mRNA level of IL-1β (**b**), TNF-α(**c**), IL-6 (**d**), IL-17A (**e**), IL-17C (**F**), IL-17F (**g**), IL-22 (**h**), IL-23a (**i**), CXCL1 (**j**), and CCL20 (**k**) in the skin biopsies from mice. Symbols defined in legend apply to **b**–**k** panels in the figure. Results are representative of three independent experiments. Error bars represent mean ± standard deviation (SD). **p < 0.01 and ***p < 0.001 when compared.

Immunohistochemistry and western blot were used to investigate the effect of inhibition of RIPK1 on necroptosis in vivo, and examination of the expression of key proteins in programmed necrosis. Immunohistochemical analysis showed that RIPK1, pRIPK3, pMLKL, and MLKL were significantly expressed in the epidermal layers in the IMQ group, while RIPK1, pRIPK3, pMLKL, and MLKL were significantly reduced with Nec-1s administration (Figs. 3e and S3). Although RIPK3 tended to decrease in the IMQ + Nec-1s group, there was no difference recorded between the two groups (Fig. S3B). Western blot analysis showed that RIPK1, pRIPK3, RIPK3, MLKL, pMLKL, and HMGB1 levels were significantly higher in IMQ group than in control group. However, this expression was significantly suppressed by Nec-1s (Figs. 3f and 4a).

To appraise the effects of Nec-1s on IMQ-induced local and systemic inflammation, the expression of proinflammatory cytokines in the skin lesions of mice was investigated. As shown in Fig. 4B–K, the mRNA level of IL-1β, TNF-α, IL-6, IL-17A, IL-17C, IL-17F, IL-22, IL-23a, CXCL1, and CCL20 was significantly decreased in the Nec-1s group compared to those in the IMQ group. These results indicate that Nec-1s pretreatment can significantly reduce the clinical manifestations and inflammatory response of IMQ mice.

### Nec-1s affects programmed necrosis rather than apoptosis in IMQ-induced psoriasiform dermatitis in mice

**Fig. 5:**
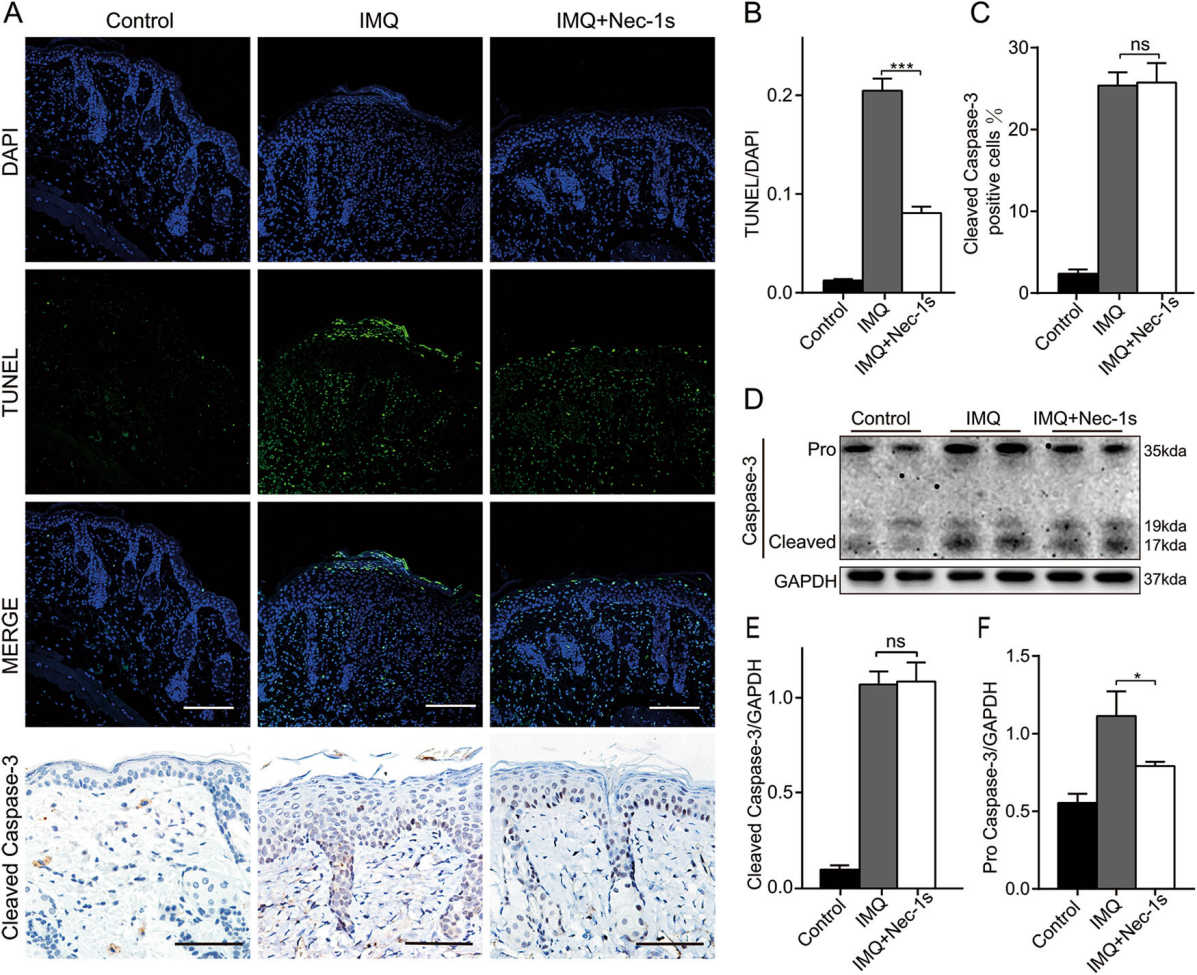
Nec-1s affected programmed necrosis rather than apoptosis in IMQ-induced psoriasiform dermatitis in mice. **a** TUNEL-stained (green fluorescence) cells in the skin biopsies from mice, co-stained with DAPI (blue fluorescence) to detect the nucleus. Immunohistochemical staining for cleaved caspase-3 in the dorsal mouse skin samples of the three groups. Representative images are shown. All scale bars represent 200 μm. TUNEL and cleaved caspase-3-positive cells were quantified under four randomly optical fields per section and normalized over the visual area counted. Four measurements were performed on each field. **b** The ratio of microscopic quantification of TUNEL-positive cells in three groups. **c** The ratio of microscopic quantification of cleaved caspase-3-positive cells in epidermis of three groups. **d** Protein level of caspase-3 was analyzed by western blotting in the three groups of mice. GAPDH served as the loading control. **e**, **f** Expression ratio of cleaved caspase-3 and pro caspase 3 to GAPDH was calculated from the relative optical density. Results are representative of three independent experiments. Error bars represent mean ± standard deviation (SD). ns *p* > 0.05, *p < 0.05, and ***p < 0.001 when compared.

RIPK1 is involved in different cell death pathways, including apoptosis and necroptosis. To distinguish between apoptotic and necrotic cells in the dorsal lesions of psoriatic mice, a dual-labeled apoptosis assay was demonstrated: Terminal deoxy nucleotidyl transferase dUTP nick end labeling (TUNEL) and immunohistochemical labeling of active caspase-3. TUNEL can recognize DNA fragmentation, a feature of both apoptotic and necrotic cells while cleaved caspase-3, an active form of caspase-3 can indicate apoptotic cells. Hence necrotic cells are considered to be positive for TUNEL staining but negative for cleaved caspase-3^23^. As shown in Fig. 5a–c, the proportion of both TUNEL-positive epidermal cells and cleaved caspase-3-positive cells in the IMQ group was significantly higher than that in the control group. TUNEL-positive cells in Nec-1s group were significantly less than those in IMQ group. However, Nec-1s almost had no influence on the percentage of cleaved caspase-3 positive cells (Fig. 5a, c). Consistent with the immunohistochemistry results, western blot analysis showed the protein level of cleaved caspase-3 in the IMQ group was significantly higher than that of the control group, but there was no significant difference from the Nec-1s group (Fig. 5d–f). Therefore, we can conclude that Nec-1s reduced the number of necroptosis cells rather than apoptotic cells in the epidermis of IMQ-induced psoriasiform dermatitis in mice.

### Comparison of the inhibitory effect of Nec-1s, GSK’872, and NSA on necroptosis in vitro

**Fig. 6:**
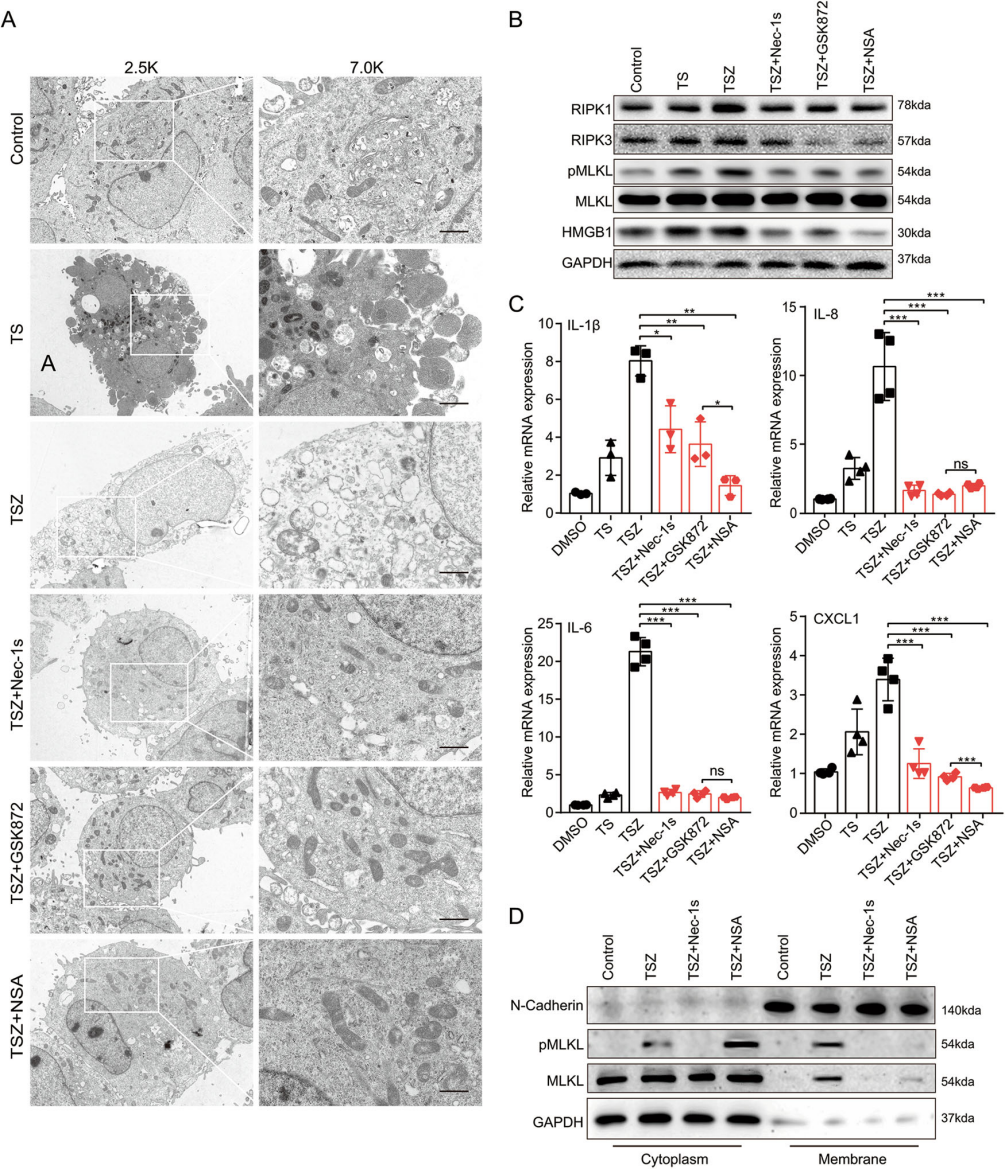
Comparison of the inhibitory effect of Nec-1s, GSK’872 and NSA on necroptosis in vitro. **a**–**c** HaCaT cells were treated with TNF-α (100 ng/mL) and Smac (100 nM) for 8 h to induce apoptosis (hereafter abbreviated as TS). Necroptosis of HaCaT cells was induced by TNF-α (100 ng/mL), Smac (100 nM), and z-VAD-fmk (20 μM) for 8 h (hereafter abbreviated as TSZ). The necroptosis cells were treated separately with the RIPK1 inhibitor Nec-1s (10 μM), the RIPK3-inhibitor GSK’872 (10 μM), and the MLKL-inhibitor NSA (5 μM). **a** Representative transmission electron microscopy (TEM) pictures of HaCaT cells treated as above. Representative images are shown. Scale bars represent 1 μm. **b** Protein levels of RIPK1, RIPK3, MLKL, pMLKL (S358) and HMGB1 were analyzed by western blotting. GAPDH was used as a loading control. **c** The mRNA level of IL-1β, IL-6, IL-8, and CXCL1 was analyzed by real-time quantitative PCR. **d** MLKL oligomerized in membrane faction upon necrosis induction. The cells were harvested and then separated into cytoplasmic and membrane proteins. Protein levels of MLKL and pMLKL (S358) were analyzed by western blotting. GAPDH was used as cytoplasm protein control. N-cadherin was used as membrane protein control. Results shown are representative data of three independent experiments. Error bars represent mean ± standard deviation (SD). ns *p* > 0.05, *p < 0.05, **p < 0.01, and ***p < 0.001 when compared.

To probe the effects of inhibiting different steps of the necroptotic signal pathway, TSZ-induced programmed necrotic cells investigated using different inhibitors. Transmission electron microscopy (TEM) is the most powerful morphological method to describe the ultrastructural changes of cells and organelles, since it can distinguish different morphological changes of apoptotic and necrotic cells^24^. TEM analysis showed that TNF-α + Smac induced cells presented morphological signs of apoptosis including cytoplasm condensation, chromatin pyknosis, and apoptotic bodies, while plasma membrane permeabilization, translucent cytosol, and swollen organelles were detected in HaCaT cells undergoing necroptosis induced by TSZ (Fig. 6a). When the RIPK1 specific inhibitor Nec-1s, RIPK3-inhibitor GSK2399872A (GSK'872), and MLKL-inhibitor NSA were administered separately to block necroptosis, cells returned to normal morphology (Fig. 6a). Furthermore, Nec-1s, GSK’872 and NSA treatment significantly reduced the levels of RIPK1, RIPK3, pMLKL, and MLKL, while NSA performed a litter better than Nec-1s and GSK’872 on reducing the expression levels of HMGB1, IL-1β, and CXCL1 (Fig. 6b, c). Indeed, NSA can bind MLKL and prevent plasma membrane rupture but has no effect on the phosphorylation of MLKL. As shown in Fig. 6d, when the HaCaT cells were treated with necroptosis inducing agents, the pMLKL signal appeared and was transferred to the membrane. Nec-1s blocked the phosphorylation signal of MLKL, while NSA blocked the transfer of pMLKL.

### NSA achieved more benefits than Nec-1s on reducing inflammation in mice with IMQ-induced psoriasiform dermatitis

**Fig. 7:**
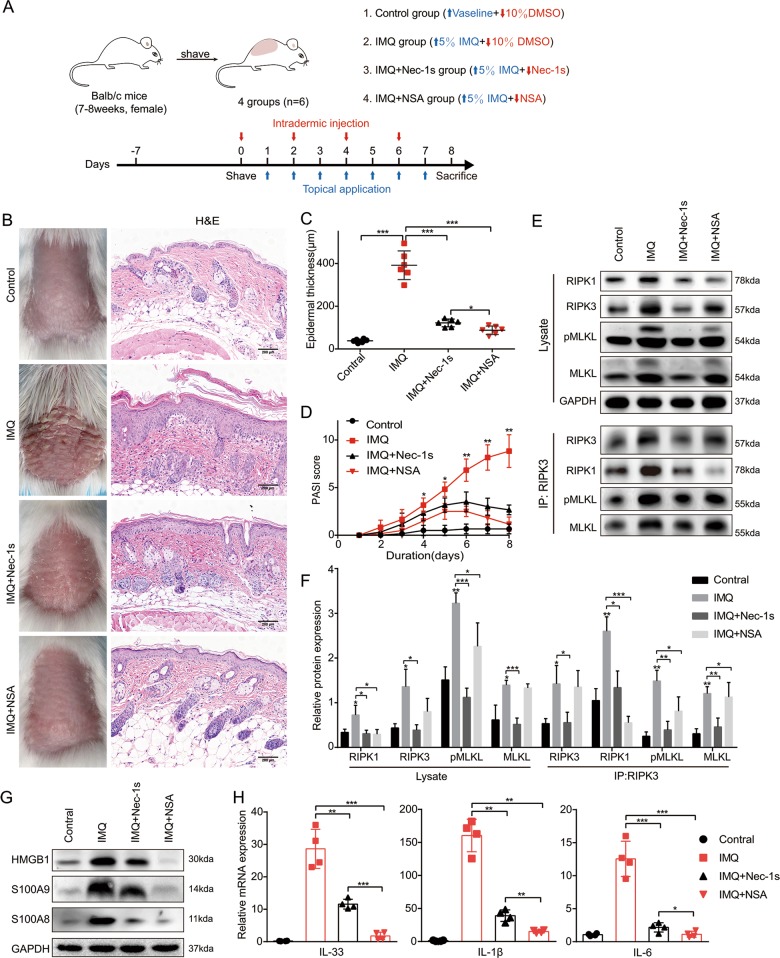
NSA achieved more benefits than Nec-1s on reducing inflammation in mice with IMQ-induced psoriasiform dermatitis. **a** Schematic representation of the animal experiment protocol for the Control, IMQ, IMQ + Nec-1s and IMQ + NSA groups (*n* = 6). **b** Representative macroscopic images taken on the 8th day and H&E staining of cross-sectional slices of the dorsal skin of BALB/c mice following continuous treatment for 8 days. **c** Epidermal thickness of the dorsal skin on the 8th day was calculated on four randomly selected optical fields per section of each mouse. **d** Daily assessment of epidermal erythema, scaling and thickening of the dorsal skin. PASI score was calculated by adding the scores of the three separate criteria (range from 0 to 12). **e**, **f** Protein levels of RIPK1, RIPK3, MLKL and pMLKL (S345) were analyzed by western blotting. GAPDH was used as a loading control. The whole lysates in the four groups was immunoprecipitated with anti-RIPK3 antibody, followed by immunoblotting of RIPK1, RIPK3, MLKL, and pMLKL (S345). **g** Protein levels of HMGB1, S100A8 and S100A9 were analyzed by western blotting. **h** Real-time quantitative PCR analysis was performed to determine the mRNA expression levels of IL-33, IL-1β, and IL-6 in skin biopsies from mice. Results shown are representative data of three independent experiments. Error bars represent mean ± standard deviation (SD). *p < 0.05, **p < 0.01, and ***p < 0.001 when compared.

Considering that RIPK3-inhibitor GSK'872 may induce undesirable effects^15^ in vivo, the anti-inflammatory effect of MLKL-inhibitor NSA by blocking necroptosis pathway and its protective effect on psoriasis compared with Nec-1s was further studied. IMQ-induced psoriasiform dermatitis mouse model and simultaneous pretreatment of Nec-1s or NSA were conducted to compare the anti-inflammatory effects of Nec-1s and NSA in vivo (Fig. 7a). Compared with the IMQ group, pretreatment with either Nec-1s or NSA significantly attenuated clinical phenotype of IMQ-induced psoriasiform dermatitis, but both did not completely eliminate the macroscopic clinical symptoms, which included redness and thickening of the skin (Fig. 7b). Histological results showed both Nec-1s and NSA significantly improved the histologic changes caused by IMQ, while NSA achieved better performance showing less parakeratosis and less inflammatory cell infiltration than the Nec-1s group (Fig. 7b). Similarly, the NSA group showed less epidermal thickening compared with the Nec-1s group (Fig. 7c). Interestingly, PASI scores were lower in the NSA group than in the Nec-1s group but there was no statistically significant difference between the two groups (*p* = 0.22) (Fig. 7d).

In the necroptosis pathway, MLKL acts as the executor protein, which functions downstream of RIPK1 and RIPK3. Therefore, NSA which targets the N-terminal coiled-coil domain of MLKL does not affect the phosphorylation by active-RIPK3^25^. Consistent with previous findings, the RIPK3 and pMLKL signal was blocked by Nec-1 rather than NSA while the formation of RIPK1-RIPK3-pMLKL complex was reduced in the two groups compared with the IMQ group (Figs. 7e, f and S4A).

Massive DAMPs released from the necroptotic cells trigger inflammatory response^26^. Necroptosis can be specifically blocked by Nec-1s and NSA, thereby reducing DAMPs including HMGB1, S100A9, and IL-33 release. Although NSA administration did not reduce the level of MLKL and p-MLKL similar to Nec-1s, NSA but not Nec-1s almost completely terminated the production of HMGB1, S100A8, S100A9, and IL-33 due to the inhibition of MLKL function (Fig. 7g, h). This suggested that there may be other cell death patterns that are independent of RIPK1 but dependent of MLKL in the IMQ mouse model. In addition, the expression level of TLR4 in NSA group was lower than that in Nec-1s (Fig. S4B). More importantly, the mRNA expression of proinflammatory cytokines IL-1β, IL-6, CCL20, IL-8, IL-17A, IL-17C, IL-17F, IL-22, IL-23a, TNF-α, and CXCL1 was significantly decreased with Nec-1s or NSA pretreatment compared to the IMQ group (Figs. 7h, and S4C–K). Therefore, NSA achieved more benefits in terms of anti-inflammatory than Nec-1s.

## Discussion

The active RIPK1 and necroptosis have been involved in many human diseases such as multiple sclerosis, myocardial ischemia-reperfusion injury, inflammatory bowel disease and Alzheimer’s disease^9^. The function of necroptosis in the pathogenesis of psoriasis seems to be complicated and its more potential mechanisms need to be further explored. Programmed necrosis-related proteins are mainly expressed and localized in the epidermis, especially in keratinocytes (Fig. 1). Immunofluorescence of TUNEL and immunohistochemical labeling of active caspase-3, RIPK1, and MLKL (Figs. 3 and 4) suggest that necroptosis mainly occurs in the upper epidermis, which is similar to the results in psoriasis patients. Certainly, we cannot exclude the possibility that necroptosis in other cells other than keratinocytes might be involved in the psoriatic pathogenesis. Our study demonstrated the presence of programmed necrosis in psoriasis, and inhibiting necroptosis can significantly reduce inflammation in vivo, causing the improvement of clinical phenotypes and the reduction of proinflammatory factors.

Disintegrated necroptotic cells release their contents to the surroundings, and these contents act as DAMPs to stimulate the surrounding inflammatory reaction. HMGB1 has a wide range of immunological activities^22^, either by itself or in combination with receptors which induces cytokine production, promotes angiogenesis, and plays an important role in autoimmune and inflammatory diseases. It is significantly increased in patients with psoriasis^27^. Our previous study found that HMGB1 is highly expressed in the psoriatic-like mouse model, upregulating the toll-like receptor 4 (TLR4) and the receptor for advanced glycation end products (RAGE) hence promoting the production of downstream inflammatory factors^28^. The S100A8/S100A9 complex is the most abundant DAMP in many autoimmune diseases. Further, proteomic analysis of the human psoriasis epidermis found thatthe complex is the most upregulated protein^29^, and recent studies have found that S100A9 is essential for the local manifestations of psoriatic inflammation^30^. The necroptotic keratinocytes in lesional psoriatic epidermis partly contribute to the localized distribution of S100 protein. The proinflammatory effect of necroptosis is not only due to cell disintegration and the release of DAMPs, but also RIPK1, RIPK3, and MLKL have been implicated in the activation of inflammasomes, which are responsible for the CASP1-dependent maturation and secretion of IL-1β and IL-18^31–33^. Moreover, activation of A Disintegrin And Metalloprotease (ADAMs) induced by pMLKL promotes the dropping-off of different cell surface proteins when the plasma membrane remains intact in the early stage of programmed necrosis^34^. Currently, more than 100 cell surface proteins have been identified as substrates for ADAM, including adhesion proteins (e.g., E-cadherin and EpCAM), cytokines (e.g., TNF-α, Fas ligand), and cytokine receptors (e.g., IL-6R and TNF-R), which promote inflammation. This partly shed light on how necroptosis directly induces inflammation. RIPK3-mediated proinflammatory is generally thought to be dependent on necroptosis. However, RIPK3 has been found to promote neutrophil recruitment by inducing cytokine and chemokine expression in keratinocytes in a necroptosis-independent manner, thereby mediating psoriasis inflammation in mice^15^. RIPK3 also promotes the formation of NLRP3 inflammasome and IL-1β inflammatory responses independent of its substrate MLKL in mice lacking inhibitor of apoptosis proteins (IAPs)^32^. The mechanism of RIPK3 promotes inflammation independent of MLKL and necroptosis remains unclear. In view of this, this study did not compare the anti-inflammatory effects of GSK’872 with the other 2 inhibitors in vivo.

The reason why NSA achieved more benefits than Nec-1s on anti-inflammation in mice was unclear. One possible explanation is the presence of RIPK1-independent necroptosis in psoriasis. In addition to RIPK1, there are two other activators in mouse and human cells, which physically interact with RIPK3 through RHIM driving necroptosis, including toll-like Receptor adaptor molecule 1 (TICAM1; also known as TRIF)^35^ and Z-DNA binding protein 1 (ZBP1)^36^. TRIF is an adaptor in the signal transduction cascade triggered by toll-like receptors (TLRs), and our previous study also found that TLR4 is elevated in the psoriasis mouse model^28^. NSA group did have lower TLR4 expression level than Nec-1s group (Fig. S4B). ZBP1 is a cytosolic DNA sensor mediating necroptosis induced by IFN-β and IFN-γ^37^, which are highly expressed in psoriasis. This suggested the possibility that multiple stimuli inducing programmed necrosis synergistically promote inflammation in the same disease. One recently characterized example is that IFN-γ and TNF-α induce necroptosis in keratinocytes, resulting in interface dermatitis like lichen planus and cutaneous lupus^38^.

This study indicated that necroptosis in keratinocytes is an important trigger of psoriatic inflammation, but the causal relationship between necroptosis and psoriasis is more like a chicken-and-egg situation^39^ which needs further study. Necroptosis provokes the inflammatory response in psoriasis, while epidermal barrier disrupted by inflammation of psoriasis makes keratinocytes exposed to multiple noxious stimuli leading to extensive necroptosis of cells. It cannot be ignored that inhibition of programmed necrosis with Nec-1s or NSA is beneficial for the treatment of psoriasis. An oral small-molecule inhibitor of RIPK1 kinase GSK2982772, which has stronger inhibitory effect and superior pharmacokinetic properties than Nec-1s potently binds to RIPK1 and excellently blocks many TNF-dependent cellular responses. It is currently in phase 2a clinical studies for psoriasis^40^. Ongoing research in this area, including discovery of new inhibitors and identification of new regulatory molecules and mechanisms, may provide new approaches to the treatment of psoriasis by targeting necroptosis.

## Materials and methods

### Chemicals and reagents

TNF-α (210-TA-005), Smac (789-SM-100), z-VAD-fmk (FMK001), Necrostatin-1s (Nec-1s), and Necrosulfonamide (NSA) were purchased from R&D (Minneapolis, MN, USA). DMSO were obtained from Sigma. GSK’872 was obtained from MCE. Following primary antibodies were used: HRP-linked anti-mouse IgG (7076 S) and anti-rabbit IgG (7074 S), Cleaved Caspase-3 (9664 S) and RIPK1 (3493 S) (Cell Signaling Technology, Danvers, MA, USA). Caspase-8 (sc-5263), TLR4 (sc-293072) and RIPK3 (sc-374639) (Santa Cruz, CA, USA). HMGB1 (ab79823), RIPK3 (ab56164), pRIPK3(S227, ab209384), pRIPK3 (S232, ab195117), MLKL (ab194699), pMLKL (S345, ab196436) and pMLKL (S358, ab187091) (Abcam, Cambridge, UK). β-actin and GAPDH (Affinity Biosciences, USA), N-cadherin (13769-1-AP) and S100A8 (15792-1-AP) (Proteintech, Rosemont, IL, USA). S100A9 (NB110-89726, Novus Biologicals, USA). Cell Counting Kit-8 (CCK-8) and cytotoxicity LDH assay kit were purchased from Dojindo China Co., Ltd (Shanghai, China). Minute ^TM^ Plasma Membrane Protein Isolation and Cell Fractionation Kit was purchased from Invent Biotechnologies, Inc. (Minnesota, USA).

### Human sample collection

Skin samples were obtained through surgical biopsy from Union Hospital, Tongji Medical College of Huazhong University of Science and Technology in Wuhan, China. All patients involved signed informed consent. The research protocols conformed to the declaration of Helsinki Principles and were approved by the medical ethics committee of Tongji Medical College of Huazhong University of Science and Technology.

### Mice

Female BALB/c-Mice (18–20 g) at 6–8 weeks of age were purchased from Beijing Huafukang Biological Technology Co., Ltd (Beijing, China). The animals were fed with a standard laboratory diet and water under specific conditions (12-h light-dark rhythm, 23 ± 2 °C ambient temperature). They were acclimatized for at least 7 days before use. All experimental protocols were conducted in accordance with the ARRIVE guidelines and were approved by the animal care and use committee of Tongji Medical College of Huazhong University of Science and Technology (Wuhan, China).

### Experimental psoriasis mouse models

BALB/c mice were randomized into several groups (*n* = 6). The mice were subjected to imiquimod-induced psoriasis-like inflammations, which were treated locally with a 42 mg daily dose of IMQ (5%) cream (Mingxin Pharmaceuticals, Sichuan, China) on the shaved dorsal skin for 7 consecutive days. Vaseline jelly was used as the control. Nec-1s and NSA were dissolved in 10% dimethyl sulfoxide (DMSO, Sigma, USA). Mice were injected intradermally with Nec-1s (1.5 mg/kg/day), NSA (50 μg/kg/day) or an equal volume of 10% DMSO (control group) every other day. Other details were described in Fig. 3a and Fig. 7a. The entire experiment was conducted in duplicate. Psoriasis Area and Severity Index (PASI) scores of mouse back skin inflammation severity was assessed by two by two laboratory assistants who did not know the group information according to previous description^41^. On day 8, serum samples and skin tissues were taken from the sacrificed mice for subsequent studies.

### Histology and immunohistochemistry (IHC) staining

Ten percent formalin-fixed, paraffin-embedded tissue sections (5 μm thickness) mounted on glass slides were used for various staining. Some sections were stained with Hematoxylin-eosin (H&E) used for evaluation of epidermal thickness. For immunochemistry, some skin paraffin sections were stained with anti-Cleaved Caspase-3 (1:150), anti-RIPK1 (1:50), anti-RIPK3 (1:500), anti-pRIPK3(S232) (1:300), anti-pMLKL(S358) (1:50), anti-pMLKL(S345) (1:500), and anti-MLKL (1:200). The pathological image was taken with the upright microscope (Olympus, Japan), and analyzed by ImageJ software. Means of epidermal thickness was calculated based on four randomly selected fields of view per mouse as previously described^28^.

### Cell culture and retroviral transduction

HaCaT cell line was purchased from the China Center for Type Culture Collection (Wuhan, China). The HaCaT cells were cultured in Minimum Essential Medium with Earle’s Balanced Salts (MEM/EBSS) (HyClone, USA) supplemented with 10% fetal bovine serum (FBS) and 1% penicillin and streptomycin (Gibco, NY, USA) in a humidified incubator with 5% CO_2_ at 37 °C. To generate cell lines with stable gene knockdown, lentivirus containing shRNA targeting RIPK1 sequence or a control sequence (from Genechem Corporation, Shanghai, China) were transfected into HaCaT cells*.* RIPK1-targeting sequence: 5′-CCACTAGTCTGACGGATAA-3′, and control sequence: 5′-TTATCCGTCAGACTAGTGG-3′. Cell lines stably expressing shRNA constructs were selected by puromycin (2 μg/ml). Knockdown efficiency was determined by detecting the level of mRNA or protein.

### In vitro stimulation of cells and analysis

HaCaT cells were treated with 100 ng/ml TNF-α, 100 nM Smac, and 20 μM z-VAD-fmk to induce necroptosis. Cells treated with DMSO served as the control. The necroptosis cells were separately treated with the RIPK1 inhibitor Nec-1s (10 μM), the RIPK3-inhibitor GSK872 (10 μM), and the MLKL-inhibitor NSA (5 μM).

Cell fixation, cell slides, and transmission electron microscopy (TEM) imaging were performed by ServiceBio (Wuhan, China). Cell viability was determined using Cell Counting Kit-8 (CCK-8) and cytotoxicity LDH assay kit according to the manufacturer’s protocols.

### Immunofluorescence

The TUNEL assay was performed on mouse skin sections using the In-Situ Cell Death Detection Kit-POD (Roche, USA) according to the manufacturer’s recommendations. Nuclei were stained with DAPI and the fluorescence images taken with confocal laser scanning microscope (Olymbus, Japan) and analyzed by ImageJ software.

### Western blotting analysis

Total protein samples from cells or tissues were extracted using cell lysis buffer for 30 min and the lysates were centrifuged at 12,000 rpm for 15 min at 4 °C. Cytoplasm and membrane protein were isolated by Minute^TM^ Plasma Membrane Protein Isolation and Cell Fractionation Kit (Invent Biotechnologies, Eden Prairie, MN, USA) according to the manufacturer’s instructions. The above protein detected by western blotting was performed according to the method described in our previous study^28^. The Abs used in this study were as follows: GAPDH (1:1000), anti-Cleaved Caspase-3 (1:1000), anti-RIPK1 (1:1000), anti-RIPK3 (1:1500), pRIPK3(S227) (1:500), anti-pRIPK3(S232) (1:1000), anti-pMLKL(S358) (1:300), anti-pMLKL(S345) (1:1000), anti-MLKL (1:1000), anti-HMGB1 (1:1000), anti-S100A8 (1:400), anti-S100A9 (1:1000), anti-TLR4 (1:300), anti-mouse IgG (1:3000), and anti-rabbit IgG (1:3000). Relative protein expression levels were assessed by the quantification of relative optical density with ImageJ software.

### Co-immunoprecipitation

Tissues of mouse back skin samples were lysed with pre-chilled IP buffer, and the supernatant was collected by centrifugation as described previously^28^. Part of the lysate was saved as input. Protein A/G agarose beads (Santa Cruz, CA, USA) were added to the remaining lysis content and incubated for 1 h at 4 °C. Supernatant was collected by centrifugation. 1.0 μg anti-RIPK3 (1:300; Santa Cruz, CA, USA) was added and incubated at 4 °C overnight, and normal mouse IgG as a negative control. Protein A/G agarose beads were again added and incubated for 4 h. The immunoprecipitated complexes were collected by centrifugation and washed four times with 1 ml pre-chilled IP buffer, followed by 40 μl loading buffer. Western blot analysis was performed on the samples as previously described.

### RNA extraction and real-time quantitative PCR

Total mRNA was isolated from cells and tissues with RNAiso plus (Takara Biotechnology, Ohtsu, Japan) according to the manufacturer’s protocols. Then the total mRNA was retro-transcribed to cDNA with a PrimeScript RT Master Mix Kit (Takara Biotechnology, Ohtsu, Japan). Real-time quantitative PCR was performed with TB Green Premix Ex Taq kit (Takara Biotechnology, Ohtsu, Japan). The 2^-△△CT^ method was used to quantitatively analyze the data. All PCR primers used in the study are as shown in Supplementary Table S1.

### Statistical analysis

The results are expressed as mean ± standard deviation, and analyzed by Graph Prism 7.0 software (GraphPad Software, San Diego, CA, USA). Student’s *t*-test or one-way analysis of variance (ANOVA) was used to analyze the statistics. *P*-values were considered as ns (no significant) *p* > 0.05, and significant when *p < 0.05, **p < 0.01, and ***p < 0.001.

## Supplementary information

Supplementary Figure Legends

Supplementary figure 1

Supplementary figure 2

Supplementary figure 3

Supplementary figure 4

Supplementary table 1
