# Supplementary material for: Inhibition of keratinocyte necroptosis mediated by RIPK1/RIPK3/MLKL provides a protective effect against psoriatic inflammation
Source: Cell Death Dis. 2020 Feb 19;11(2):134. doi: 10.1038/s41419-020-2328-0 (PMC7031250; doi:10.1038/s41419-020-2328-0)
Supplement: Supplementary file 1 — Supplementary Figure Legends [file 41419_2020_2328_MOESM1_ESM.docx]

**Supplementary Figure 1. Expression of necroptosis-related genes in IMQ-induced psoriasiform dermatitis in mice.**

(**A**) Representative macroscopic views and H&E staining of cross-sectional slices of the dorsal skin of BALB/c mice following continuous treatment with vaseline or IMQ for 8 days. Scale bar represents 200 μm. (**B**) Protein levels of RIPK1, RIPK3, MLKL and pMLKL(S345) of two groups were analysed by western blotting. (**C**) mRNA expression of RIPK1, RIPK3, and MLKL was analysed by real-time quantitative PCR in skin biopsies from the two groups.

Data are representative of three independent experiments. Error bars in (**C**) represent mean ± SD. *p<0.05, **p<0.01, and ***p < 0.001 compared with the control group.

**Supplementary Figure 2. Screening for the appropriate duration to induce necroptosis in HaCaT cells.**

HaCaT cells was treated with TNF-α (100 ng/mL), Smac (100 nM), and z-VAD-fmk (20 μM) (hereafter abbreviated as TSZ) for 0, 2, 4, 6, 8, 10, 12 hours respectively. (**A**) Cytotoxicity LDH assay kit was used to detect the LDH release rate of TSZ-treated HaCAT cells. (**B**) Cell viability of TSZ-treated HaCAT cells was measured by the Cell Counting Kit-8 (CCK-8) assay. (**C**) Protein levels of pMLKL(S358) and HMGB1were analyzed by western blotting.

Data are representative of three independent experiments. Error bars in (A-B) represent mean ± SD. **p < 0.01 by when compared.

**Supplementary Figure 3. Nec-1s** **slightly affects expression of RIPK3 in IMQ-induced psoriasiform dermatitis in mice.**

(**A**) Immunohistochemical staining for pRIPK3(S232), pMLKL (S345) and RIPK3 in the dorsal mouse skin samples of controls, IMQ, and IMQ+Nec-1s groups (n=6). Representative images are shown. Scale bar represents 200 μm. (**B**) Semi-quantitative result of immunostaining for pRIPK3, pMLKL and RIPK3 in the Control, IMQ and IMQ+Nec-1 group.

Data are representative of three independent experiments. Error bars in (**B**) represent mean ± SD. **ns** p>0.05, **p < 0.01 and ***p < 0.001 when compared.

**Supplementary Figure 4. The effect of Nec-1s and NSA on the expression of key proinflammatory cytokines and chemokines** **in IMQ-induced psoriasiform dermatitis in mice.**

(**A**) The whole lysates in IMQ group was immunoprecipitated with anti-RIPK3 antibody, followed by immunoblotting of MLKL. The tissue lysates from IMQ group was used as input. Anti-mouse IgG was used as a negative control. (**B**) Protein levels of TLR4 in four groups were analysed by western blotting. Real-time quantitative PCR analysis was performed to determine the mRNA expression levels of CCL20 (**C**), IL-8 (**D**), IL-17A (**E**), IL-17C (**F**), IL-17F (**G**), IL-22(**H**), IL-23a (**I**), TNF-α (**J**), and CXCL1 (**K**) in skin biopsies from mice. Symbols defined in legend apply to **C**-**K** panels in the figure.

Results are representative of three independent experiments. Error bars in (**C-K**) represent mean ± standard deviation (SD). ns p>0.05, *p<0.05, **p<0.01, and ***p < 0.001 when compared.
