# Supplementary figures and images for: Inhibition of keratinocyte necroptosis mediated by RIPK1/RIPK3/MLKL provides a protective effect against psoriatic inflammation
Source: Cell Death Dis. 2020 Feb 19;11(2):134. doi: 10.1038/s41419-020-2328-0 (PMC7031250; doi:10.1038/s41419-020-2328-0)

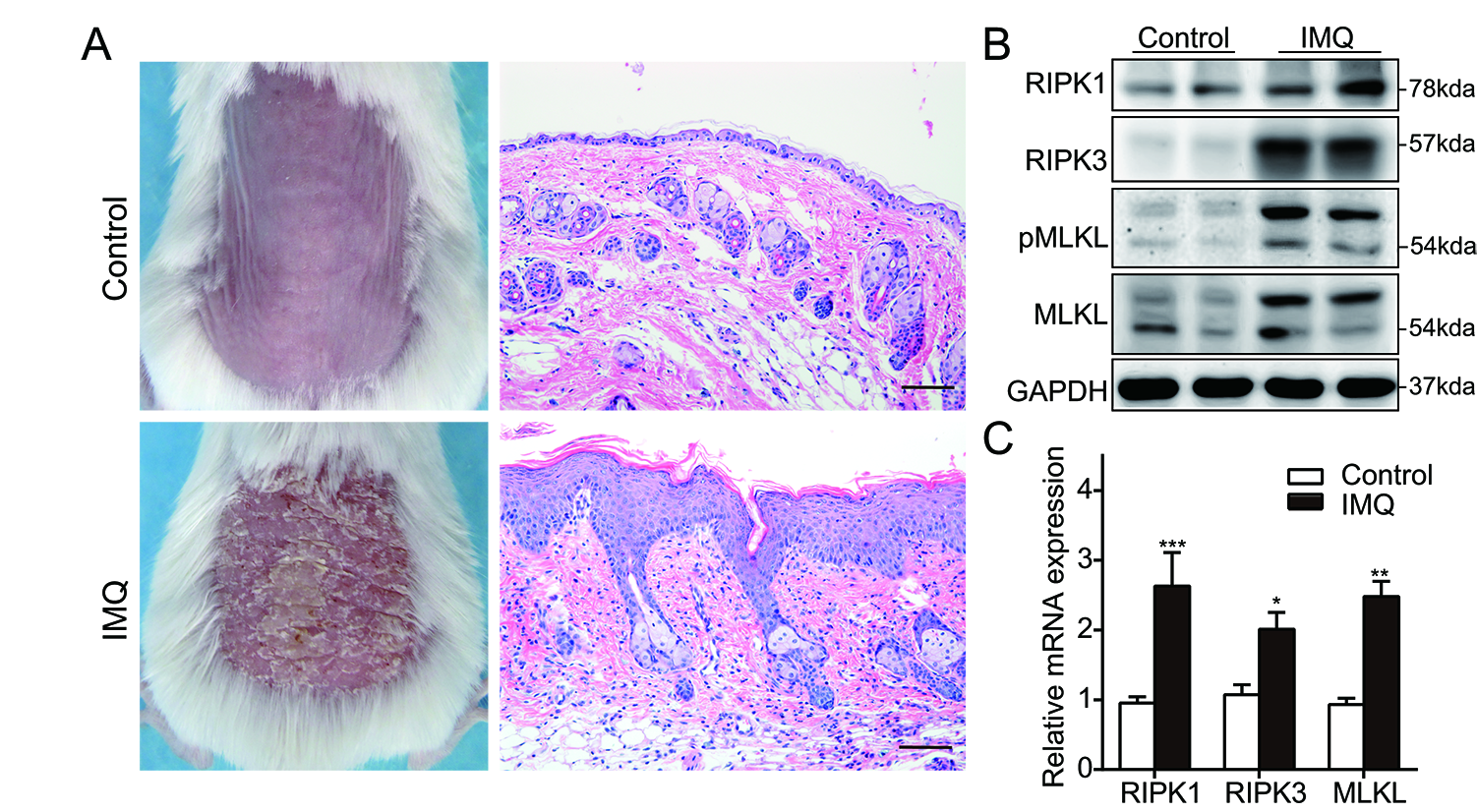

Supplement: Supplementary file 2 — Supplementary figure 1 [file 41419_2020_2328_MOESM2_ESM.tif]

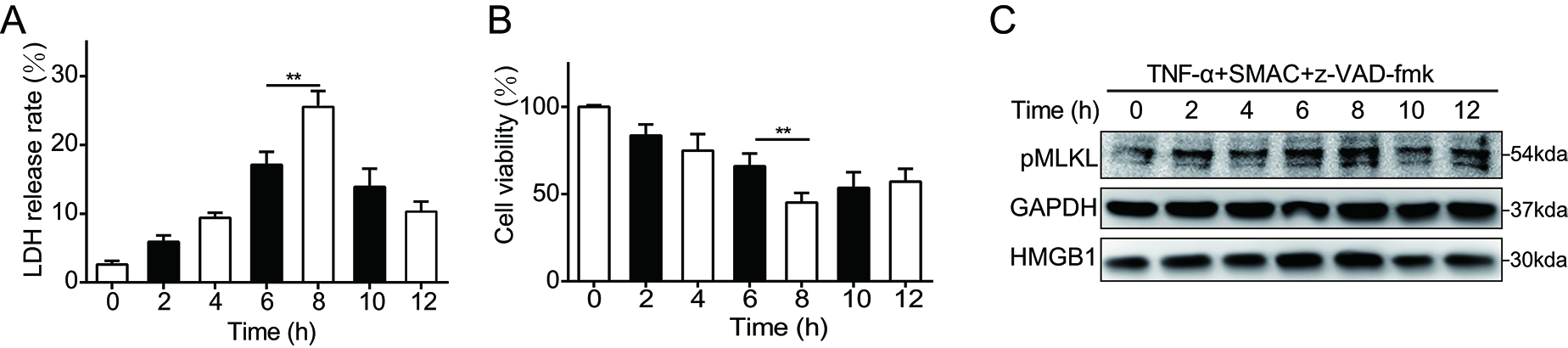

Supplement: Supplementary file 3 — Supplementary figure 2 [file 41419_2020_2328_MOESM3_ESM.tif]

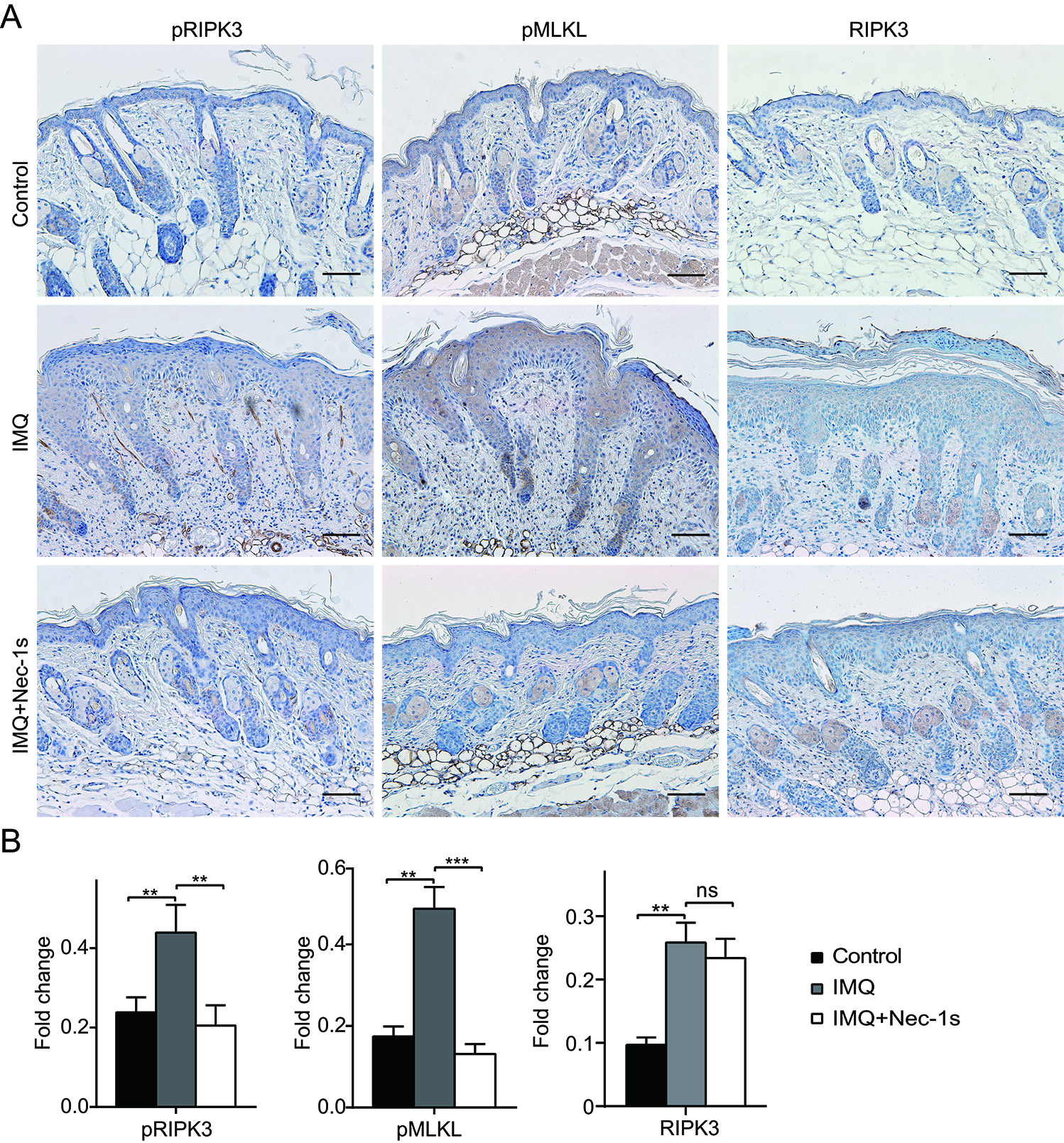

Supplement: Supplementary file 4 — Supplementary figure 3 [file 41419_2020_2328_MOESM4_ESM.tif]

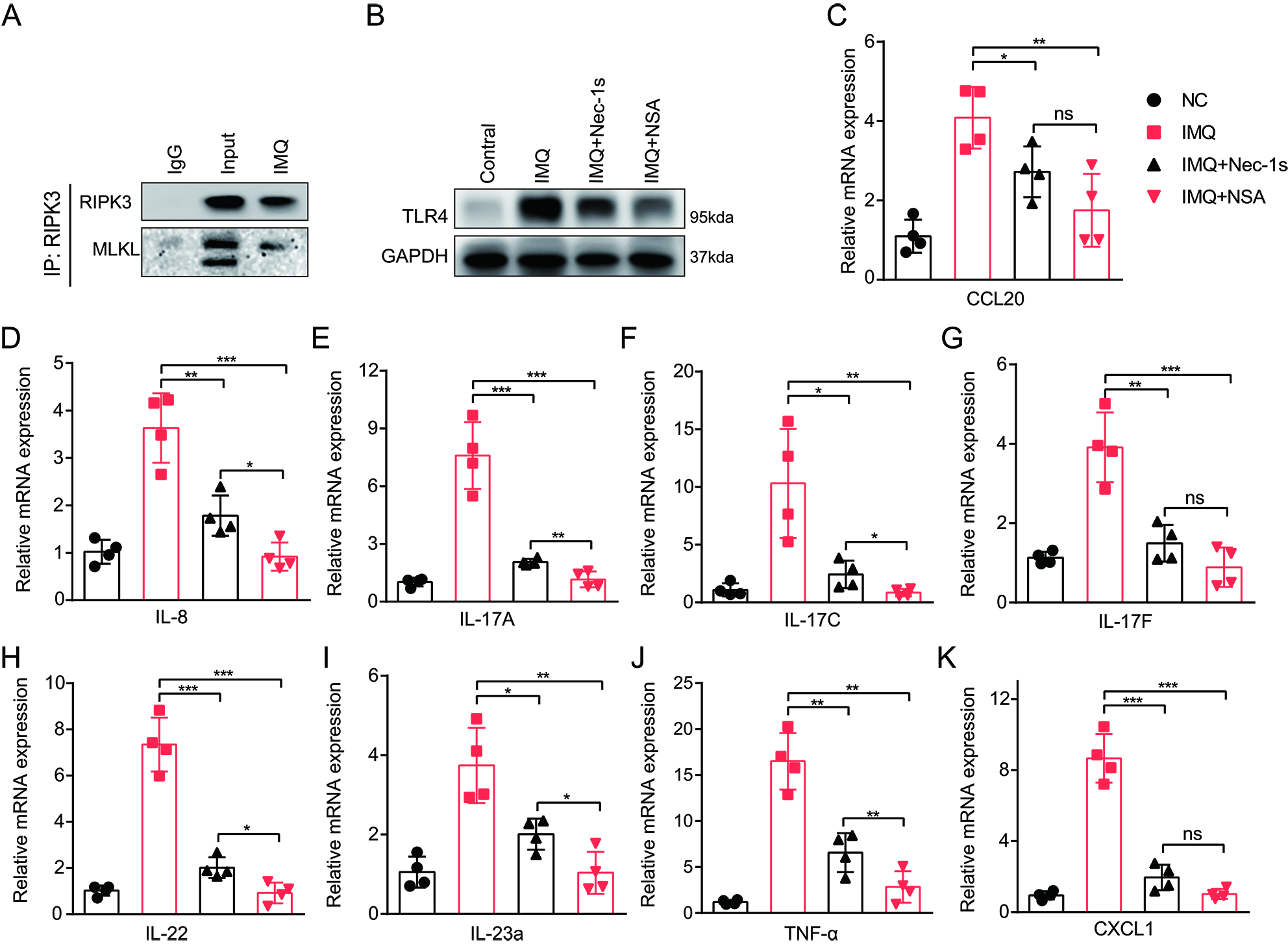

Supplement: Supplementary file 5 — Supplementary figure 4 [file 41419_2020_2328_MOESM5_ESM.tif]
