# Supplementary material for: Inhibition of keratinocyte necroptosis mediated by RIPK1/RIPK3/MLKL provides a protective effect against psoriatic inflammation
Source: Cell Death Dis. 2020 Feb 19;11(2):134. doi: 10.1038/s41419-020-2328-0 (PMC7031250; doi:10.1038/s41419-020-2328-0)
Supplement: Supplementary file 6 — Supplementary table 1 [file 41419_2020_2328_MOESM6_ESM.docx]

**Table 1. Primer sequences for real-time quantitative PCR.**

|  | Forward 5’-3’ | Reverse 5’-3’ |
| --- | --- | --- |
| Homo RIPK1 | GGGAAGGTGTCTCTGTGTTTC | CCTCGTTGTGCTCAATGCAG |
| Homo RIPK3 | ATGTCGTGCGTCAAGTTATGG | CGTAGCCCCACTTCCTATGTTG |
| Homo MLKL | AGGAGGCTAATGGGGAGATAGA | TGGCTTGCTGTTAGAAACCTG |
| Homo IL-1β | ATGATGGCTTATTACAGTGGCAA | GTCGGAGATTCGTAGCTGGA |
| Homo IL-6 | ACTCACCTCTTCAGAACGAATTG | CCATCTTTGGAAGGTTCAGGTTG |
| Homo IL-8 | ATGACTTCCAAGCTGGCCGTGGCT | TCTCAGCCCTCTTCAAAAACTTCTC |
| Homo CXCL1 | TGCTGTACCAAGAGTTTGCTC | CGCACACAGACAACTTTTTCTTT |
| Homo GAPDH | GGCTCTCCAGAACATCATC | TCTTCCTCTTGTGCTCTTG |
| Mus RIPK1 | AGAAGAAGGGAACTATTCGCTGG | CATCTATCTGGGTCTTTAGCACG |
| Mus RIPK3 | CAGTGGGACTTCGTGTCCG | CAAGCTGTGTAGGTAGCACATC |
| Mus MLKL | TTAGGCCAGCTCATCTATGAACA | TGCACACGGTTTCCTAGACG |
| Mus GAPDH | TCTCCTGCGACTTCAACA | TGTAGCCGTATTCATTGTCA |
| Mus IL-1β | CTTCAGGCAGGCAGTATC | CAGCAGGTTATCATCATCATC |
| Mus IL-6 | CCGCTATGAAGTTCCTCTC | GGTATCCTCTGTGAAGTCTC |
| Mus TNF-α | TGTCCATTCCTGAGTTCTG | GGAGGCAACAAGGTAGAG |
| Mus IL-17A | ACTACCTCAACCGTTCCA | GAATCTGCCTCTGAATCCA |
| Mus IL-17C | ATGCTTGTGTCGTGGATG | GTGCCTGGAATGTCTGTC |
| Mus IL-17F | TGCTACTGTTGATGTTGGGAC | AATGCCCTGGTTTTGGTTGAA |
| Mus IL-22 | ATGAGTTTTTCCCTTATGGGGAC | GCTGGAAGTTGGACACCTCAA |
| Mus IL-23a | ACCTGCTTGACTCTGACA | CCACTGCTGACTAGAACTC |
| Mus IL-33 | TCCAACTCCAAGATTTCCCCG | CATGCAGTAGACATGGCAGAA |
| Mus CXCL1 | CTGGGATTCACCTCAAGAACATC | CAGGGTCAAGGCAAGCCTC |
| Mus CCL20 | GCCTCTCGTACATACAGACGC | CCAGTTCTGCTTTGGATCAGC |
